# Weight management medications for chronic use in 37 veterans affairs medical centers—A medication use evaluation

**DOI:** 10.1002/osp4.70002

**Published:** 2024-08-30

**Authors:** Samantha Walczuk, Muriel Burk, Elaine Furmaga, Samaneh Ghassemi, Madeline McCarren, Kenneth Bukowski, Peter Glassman, Fran Cunningham

**Affiliations:** ^1^ Veterans Affairs (VA) Center for Medication Safety Hines Illinois USA; ^2^ United States Department of Veterans Affairs VA Pharmacy Benefits Management Services Washington District of Columbia USA; ^3^ VA Greater Los Angeles Healthcare System Los Angeles California USA; ^4^ David Geffen School of Medicine at UCLA Los Angeles California USA; ^5^ Present address: Jesse Brown VA Medical Center Chicago Illinois USA.

**Keywords:** drug utilization, health systems, obesity, obesity treatment

## Abstract

**Rationale:**

Controlled trials have demonstrated successful weight loss associated with certain weight management medications (WMMs). However, there are limited real‐world data on prescribing patterns and efficacy and safety profiles of WMMs in Veterans Affairs (VA) patients.

**Objective:**

To evaluate: utilization patterns of WMMs liraglutide, naltrexone/bupropion, orlistat, phentermine, phentermine/topiramate, and semaglutide; weight loss at three, six, twelve, and more than 12 months; safety; and treatment barriers.

**Methods:**

A retrospective, cross‐sectional medication use evaluation (MUE) was conducted using electronic health records of outpatient Veterans newly initiated on WMMs at 37 VA Medical Centers between 1 March 2020 and 31 March 2022. Chart review was used to identify WMM utilization and capture rates of clinical response, defined as 5% and 10% or greater weight loss at the final weight, adverse drug events (ADEs), non‐adherence, and discontinuations. Site‐specific surveys evaluated local practices and barriers.

**Results:**

Among 1959 eligible Veterans, semaglutide, phentermine/topiramate, and orlistat were most frequently prescribed. The clinical response was highest among phentermine/topiramate, liraglutide, and semaglutide. Naltrexone/bupropion and phentermine demonstrated the highest and lowest ADE rates, respectively. Potential barriers to WMM utilization and successful treatment by site reports were drug shortages, patient perceptions of therapeutic course, personal preferences, and VA WMM use criteria.

**Conclusions:**

Smaller weight loss and higher discontinuation rates were observed relative to clinical trials. The MUE data allow for better assessment of benefits and risks for Veterans prescribed WMMs.

## 1 INTRODUCTION

An estimated 78% of Veterans have a body mass index (BMI) in the overweight or obese categories, increasing their risk of diabetes, cardiovascular morbidity, certain cancer types, and lower quality of life.^1^, ^2^ While physical activity, diet and behavior modifications are considered first‐line treatments for obesity, patients may require Food and Drug Administration (FDA)‐approved medications to achieve weight management goals. In patients with either BMI ≥30 kg/m^2^ or BMI≥27 kg/m^2^ with obesity‐related conditions, the Veterans Affairs (VA)/DoD Clinical Practice Guideline for the Management of Adult Overweight and Obesity suggests offering weight management medications (WMMs) as adjunct therapy to comprehensive lifestyle interventions (CLI) (i.e., behavioral, dietary, and physical activity components).^1^ Within the VA, CLI is formally delivered through the MOVE! weight management program as the primary mechanism for fulfilling CLI criteria. Patients may attend an approved non‐VA CLI program, but this is not a typical occurrence.

WMMs available in the VA with Criteria for Use (CFU) at the time of the evaluation included phentermine/topiramate, orlistat, naltrexone/bupropion, liraglutide, and semaglutide. Several studies within the VA have shown significant weight loss in Veterans on WMMs.^3^, ^4^, ^5^ One retrospective analysis review of WMMs in the VA by Pendse et al. found efficacy results similar to other studies.^3^ Another VA database analysis by Grabarczyk (2018) assessed weight loss rates of orlistat, phentermine, and phentermine/topiramate when paired with the VA's MOVE! program compared with weight loss associated with the MOVE! program alone.^4^ The study showed that achievement of ≥5% weight loss after 20 or more weeks differed significantly among groups, ranging from 26.2% in the MOVE!‐only group, to 40.3% in the phentermine‐topiramate group. A retrospective cohort study also found participants in the MOVE! program in conjunction with receiving a WMM demonstrated a greater probability of reaching a weight loss of ≥5% at 6, 12, and 24 months compared to MOVE! participants without WMM use.^5^ A single‐center, retrospective chart review conducted by Hood et al. revealed weight loss rates for orlistat, liraglutide, phentermine, phentermine/topiramate, and naltrexone/bupropion similar to those found in large, controlled trials (e.g., 58.3% achieved ≥5% weight loss compared to 21%–63% in clinical trials).^6^, ^7^, ^8^, ^9^, ^10^, ^11^

While there is evidence of successful weight loss with certain WMMs among Veterans, further real‐world data are needed to assess the more recently approved semaglutide for chronic weight management. Marketed as WEGOVY® for weight loss, this drug was associated with significantly higher body weight change compared to placebo (−14.9% vs. −2.4%) and greater proportion of participants achieving ≥5% weight loss (86.4% vs. 31.5%) in phase 3 trials.^12^, ^13^, ^14^, ^15^, ^16^, ^17^ However, the mean age (46 years) and predominantly female population studied make these findings less applicable to the older and predominantly male VA population, hence, the need for investigation of WEGOVY®’s effects among Veterans.

Given the severity of certain adverse drug events (ADEs) associated with WMMs, there is a need to assess prescribing trends and observed patient experiences of WMMs in the VA. A medication use evaluation (MUE) is a systematic and interdisciplinary performance improvement tool that employs descriptive and exploratory analyses on medication utilization to identify operational needs in patient care with the goal of optimizing patient outcomes.^18^ The VA Center for Medication Safety (VAMedSAFE) conducts MUEs to assess safe and appropriate use of medications in Veterans through data abstraction of prescribing patterns and observed patient experiences. We conducted a multi‐site MUE on prescribing patterns and reported outcomes of selected WMMs at 37 VA medical centers (VAMCs) to assess the safety and extent of weight loss achieved by each WMM to provide an informed risk‐benefit assessment in a real‐world setting. This MUE evaluated prescribing and utilization patterns of WMMs, weight loss at 3, 6, 12, and >12 months while on WMMs, and the safety of WMMs. Additionally, patient‐ and provider‐specific barriers to achieving successful weight management were assessed.

## 2 METHODS

A retrospective, cross‐sectional evaluation of Veterans initiated on a WMM between 1 March 2020 and 31 March 2022 was conducted. Initiation was defined as the absence of prescriptions for any of the six selected WMMs within 12 months prior to the index date, which was the earliest release date of the first WMM prescribed during the MUE timeframe. The release date indicates when the prescription was mailed or dispensed at the pharmacy. The MUE cohort was derived from a database extraction using outpatient prescription information from the VA Corporate Data Warehouse and applying specific criteria for weight loss indication (e.g., diagnosis of Type 2 diabetes mellitus, must have BMI ≥27 kg/m^2^, semaglutide (OZEMPIC®) prescription label to include “for weight management”).^19^

Voluntary participation was solicited from all VAMCs. Site‐specific patient lists were provided to participating VAMCs (n = 37 sites). These lists contained incident users of the six WMMs (liraglutide, naltrexone/bupropion, orlistat, phentermine, phentermine/topiramate, and semaglutide (WEGOVY® and OZEMPIC®). Pharmacists and postgraduate pharmacy residents at VAMCs manually reviewed patient charts to verify eligibility and collect information.

Participating sites were instructed to follow a standardized process for chart review and submission of de‐identified data via a web‐based Microsoft InfoPath® tool through a secure SharePoint portal. A chart review goal of 100 eligible Veterans was set per site. This number was derived from a combination of feasibility and reasonably precise confidence intervals for the outcome “≥5% weight loss” based on literature estimates.^13^, ^14^, ^20^, ^21^ For each eligible case, all subsequent WMMs prescribed during the MUE timeframe were assessed to capture potential switches.

To be eligible, the Veteran must have received at least one WMM during the MUE timeframe and had a recorded baseline weight and BMI of ≥27 kg/m^2^ within 120 days prior to the index date. Review of past medical history spanned 24 months prior to the index date. Specific ADEs, medication discontinuation, and weight loss measures were assessed from the index date through WMM discontinuation or the end of the evaluation period, as applicable. Discontinuation was determined by provider documentation, any self‐reported discontinuation, or absence of subsequent prescription release dates. Temporal association of an event with no clear alternative etiology satisfied the condition for ADE attribution to the WMM as determined by the reviewer. To standardize reporting of increased heart rate (HR) and elevated blood pressure (BP) as ADEs, we pre‐defined this as HR > 100 bpm, and either systolic BP > 160 mmHg or diastolic BP > 100 mmHg without prior occurrence during the 30 days before WMM initiation.

Aggregated chart review data were used to describe prescribing and utilization patterns. The following measures were collected: baseline recorded weight and BMI prior to index date, documented participation in VA MOVE! or other approved non‐VA CLI, obesity‐related comorbidities, and use of concomitant medications associated with weight gain. While CLI primarily consists of the VA MOVE! program, reviewers could include other approved non‐VA CLI programs. Suicidal history was collected from provider notes in the 24 months prior to the index date, although the event described may have taken place before this timeframe. Information on dosing, place in therapy, and therapeutic transitions between any of the six WMMs during the MUE timeframe were collected. Proportion of Veterans remaining on each WMM was calculated for <3 months, 3 to <6 months, 6 to <12 months, or ≥12 months, along with documented adherence. Recorded weights at baseline, 3, 6, and 12 months were collected for each WMM, as applicable, including an overall final weight. Due to the SARS‐CoV‐2 pandemic necessitating virtual patient visits, we accepted self‐reported and clinic‐measured weights. Safety measures included the ADE rate for each WMM as well as discontinuation rates, inclusive of reasons. To reflect actual patient usage, medication adherence was first based on provider notation. If provider notation was missing, the reviewer calculated the Medication Possession Ratio (MPR); an MPR is calculated as the sum of days' supply dispensed in a time period divided by the days in that period and reported as a percentage. An MPR≥80% was considered adherent.

Following chart review, participant facilities completed a survey evaluating local practices and policies on WMM prescribing and drug shortage management strategies as well as patient‐ and provider‐specific barriers to the optimal use of WMMs.

VAMedSAFE conducted aggregate analysis of abstracted data. Site data were combined to produce national totals and median values, with percentages and 95% confidence intervals as appropriate. Descriptive statistics were used to report on the main variables of interest. To control for potential variation in weight measurement methods, weight differences were calculated only for matched weight documentation methods (clinic or self‐report). Across a broad range of variables, we saw no consistent trends between the groups with clinic‐measured or self‐reported weight differences, so the groups were not separated in the results. Weeks of therapy, when missing, were estimated using available dates. Due to the descriptive nature of an MUE, potential confounding effects were not addressed.

All protected health information (PHI) was maintained behind the VA firewall. The patient lists contained a crosswalk to assigned non‐identifiable IDs used for all data submission.

This MUE was deemed a quality improvement project by the Edward Hines, Jr., VA Institutional Review Board and met exemption criteria based on VA Program Guide 1200.21.^22^ All participant sites signed data use agreements.

## 3 RESULTS

**FIGURE 1:**
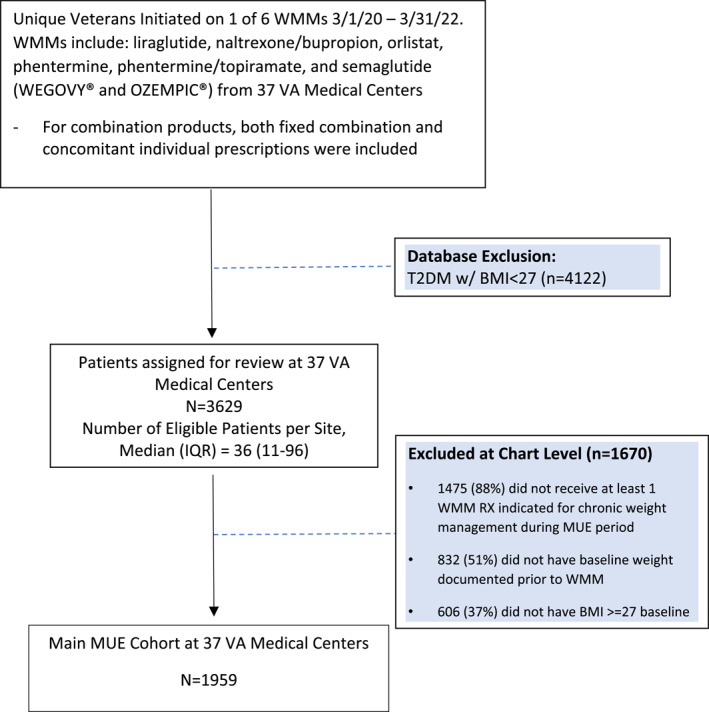
Medication use evaluation cohort flow diagram.

Thirty‐seven VAMCs participated in the MUE. Of 3629 Veterans initiated on a WMM during the MUE timeframe and assigned for review, 1670 patients (46%) were excluded, mainly due to not having chronic weight management as the primary indication for the WMM. Half of the excluded patients did not have a baseline weight recorded in the chart, and about one‐third did not have a baseline BMI ≥27 kg/m^2^. The final MUE cohort consisted of 1959 Veterans meeting eligibility criteria (Figure 1).

The eligible cohort was mostly male and Caucasian with a median age of 51 years at index date (Table 1). The most common comorbidities identified were psychiatric diagnosis (61%), hypertension (54%), obstructive sleep apnea (47%), and dyslipidemia (43%). Most patients were categorized as Class III obesity (48%), followed by 31% and 21% for Class II and Class I, respectively. The median baseline weight and BMI were 118 and 39 kg/m^2^, respectively (Table 2). Most Veterans participated in at least one visit to the MOVE! or other approved CLI program.

**TABLE 1:** Demographics and comorbidities.

Patient characteristics	*N* = 1959	%
Age (years) at time of index WMM, median (IQR)^a^	51 (42–61)	N/A
Sex		
Female	695	36
Male	1224	64
Race		
Am. Indian	22	1
Asian	25	1
Black	399	22
Hispanic	90	5
White	1313	71
Comorbidities		
Psychiatric diagnosis^b^	1204	61
Bipolar/schizophrenia spectrum and other unspecified psychotic disorders	162	8
Hypertension	1061	54
Obstructive sleep apnea	912	47
Dyslipidemia	852	43
Cancer	483	25
Prediabetes	426	22
Type 2 DM	392	20
Osteoarthritis	382	20
Metabolic syndrome	239	12
Hypothyroidism	222	11
Cardiovascular disease	186	9
Non‐opioid substance use disorder	162	8
Non‐alcoholic fatty liver disease	125	6
Arrhythmias	47	2
Cholelithiasis	31	2
Glaucoma	24	1
Hyperthyroidism	13	1
Opioid use disorder	12	1
Number of comorbidities per patient, *n* (%)	*N* = 1959	%
**0**	64	3
**1**	185	10
**2**	336	17
**3**	388	20
**4**	371	19
5	297	15
>6	318	16

^a^ IQR, Interquartile Range.

^b^ The most common psychiatric diagnoses found included anxiety, bipolar disorder, depression, psychosis, and schizophrenia. WMM use in patients with psychiatric diagnosis was of interest as there is limited data available in the literature. In light of the WMM clinical trial exclusion criteria, bipolar and psychosis disorders were evaluated as a separate subset of psychiatric diagnoses to inform on potential modifications to national criteria for use.

**TABLE 2:** Baseline weight characteristics and management strategies.

Patient characteristics	*N*	% or IQR
Baseline weight (kg), median (IQR)^a^	118	103–137
Baseline BMI (kg/m^2^), median IQR)	39	35–44
Overweight with BMI 27 to <30	54	3
Obese (BMI ≥ 30)	1899	97
Class I (BMI 30 to < 35)	395	21
Class II (BMI 35 to < 40)	589	31
Class III (BMI 40 or higher)	915	48
Lifestyle interventions		
Attended MOVE! or approved CLI	1630	88
Declined/Did not attend MOVE! or approved CLI	84	5
No mention of MOVE! or formal CLI	132	7

^a^ IQR = Interquartile Range.

The overall frequency of each prescribed WMM as well as the proportion that was prescribed as first line agents are reported (Table 3). All semaglutide (i.e., OZEMPIC® and WEGOVY®) prescriptions were combined. Patients may have received more than one type of WMM at various points during the MUE timeframe. The three most frequently prescribed WMMs overall were semaglutide (29%), phentermine/topiramate (25%) and orlistat (23%). The following highest daily (or weekly for liraglutide and semaglutide) doses were most frequently prescribed: phentermine 37.5 mg (52%); phentermine/topiramate 7.5 mg/46 mg (49%) and 15 mg/92 mg (24%); orlistat (ALLI®) 360 mg (44%), orlistat (XENICAL®) 360 mg (33%); naltrexone/bupropion 32 mg/360 mg (66%); liraglutide 3 mg (88%), semaglutide (OZEMPIC®) 1 mg (33%) and 2 mg (31%) and semaglutide (WEGOVY®) 2.4 mg (11%). The most frequently prescribed concomitant drugs with weight gain potential were antihistamines, beta‐blockers, and anticonvulsants (Table 3).

**TABLE 3:** Medications prescribed during the MUE timeframe.

WMM	Use overall (%)^a^ *N* = 1959	First line use (%^c^)
Weight management medication		
Phentermine	110 (6)	94/110 (85)
Phentermine/topiramate	490 (25)	443/490 (90)
Orlistat	441 (23)	390/441 (88)
Naltrexone/bupropion	311 (16)	269/311 (86)
Liraglutide (SAXENDA®)	347 (18)	257/347 (74)
Semaglutide (OZEMPIC® and WEGOVY®)	572 (29)^b^	401/572 (70)
Concomitant weight gain medication		
Antihistamines	359 (19)	N/A
Beta‐blocker	301 (16)	N/A
Anticonvulsant	280 (15)	N/A
Antipsychotic	186 (10)	N/A
Hormonal agents	146 (8)	N/A
Alpha‐blockers	113 (6)	N/A
Insulin	100 (5)	N/A
Tricyclic antidepressants	64 (3)	N/A
Glucocorticoids	62 (3)	N/A
Sulfonylurea	41 (2)	N/A
Thiazolidinediones	14 (0.7)	N/A

^a^ The sum of %’s may exceed 100 as patients may have received multiple WMMs during the MUE timeframe.

^b^ Of all Veterans that received a semaglutide prescription during the MUE, about 83% received the semaglutide (OZEMPIC®) formulation.

^c^ The percentage describes the proportion of Veterans who received the WMM as a first line agent among all Veterans who received the specific WMM during the MUE timeframe.

Patients who received semaglutide, liraglutide, and phentermine/topiramate had the highest proportion achieving ≥5% or ≥10% weight loss at the final weight recorded (Table 4). While there was variation in the extent of reported weight loss for each WMM, the rate of weight loss per week appeared similar across WMMs (Table 4).

**TABLE 4:** Weight (wt) loss experience^a^

	Phentermine *N* = 110	Phentermine/topiramate *N* = 490	Orlistat *N* = 441	Naltrexone/Bupropion *N* = 311	Liraglutide *N* = 347	Semaglutide *N* = 572
Total weight loss experience						
Wt loss kg/week, median (IQR)	0.1 (0–0.3)	0.2 (0.02–0.4)	0.1 (0–0.3)	0.2 (0–0.4)	0.2 (0–0.3)	0.2 (0.1–0.4)
Wt loss, −/+ kg, median (IQR)	3 (0–7)	4 (0–11)	1 (−1 to 3)	3 (0–7)	5 (0–10)	8 (2–5)
≥ 5% Wt total loss at final wt, (%)	19/97 (20)	118/448 (26)	40/398 (10)	55/273 (20)	68/266 (26)	175/426 (41)
≥ 10% Wt total loss at final wt, (%)^b^	8/66 (12)	69/303 (23)	11/246 (4)	27/211 (13)	40/206 (19)	126/359 (35)
First line therapies						
≥ 5% Wt loss at 3 months (%)^c^	12/35 (34)	80/172 (47)	14/76 (18)	34/84 (40)	59/120 (49)	104/206 (50)
≥ 5% Wt loss at 6 months (%)^c^	17/25 (68)	85/129 (66)	19/59 (32)	37/61 (61)	59/93 (63)	123/175 (70)
≥ 5% Wt loss at 12 months (%)^c^	4/7 (57)	43/57 (75)	5/23 (22)	14/23 (61)	30/49 (61)	57/75 (76)
≥ 5% Wt total loss at final wt, (%)^c^	19/62 (30)	118/280 (42)	40/233 (17)	55/187 (29)	68/164 (41)	175/279 (63)
% Wt total loss from baseline, median (IQR; N)^d^	2.5 (0.0–5.6; 66)	3.7 (0.0–9.6; 302)	0.5 (−0.5–3; 246)	2.2 (0–5.8; 211)	4 (0–8.2; 206)	7 (1.9–12.8; 359)

^a^ *N* = 2271 the unit of analysis for this table represents patient‐medication exposures since patients may have received 1 or more WMM during the MUE timeframe.

^b^ The final weight was defined as the most recent weight while the Veteran was receiving the WMM. The final weight may have occurred after the MUE timeframe.

^c^ A loss of 5% or more of weight at 3, 6, 12, and final weight was calculated for each medication when used as the index or first‐line therapy.

^d^ The median percent weight total loss from baseline was calculated for each medication when used as the index or first‐line therapy. Patients were limited to those with matched weight documentation methods.

Gastrointestinal ADEs were the most frequently reported among all medications except phentermine (Table 5). Other ADEs differed in frequency across the different WMMs. Discontinuation secondary to ADE ranged from 12% to 29%. Discontinuation for any reason, including ADEs, was most frequently documented for orlistat (80%) and naltrexone/bupropion (75%).

**TABLE 5:** Safety and persistence measures.

	Phentermine *N* = 110	Phentermine/topiramate *N* = 490	Orlistat *N* = 441	Naltrexone/Bupropion *N* = 311	Liraglutide *N* = 347	Semaglutide *N* = 572
ADE, *n* (%)	15 (14)	154 (32)	161 (37)	132 (43)	116 (34)	212 (36)
Abdominal pain					11 (8)	22 (9)
Acute cholelithiasis					1 (1)	4 (2)
Acute pancreatitis					2 (1)	
Bipolar/Psychotic symptoms		1 (1)		2 (1)	1 (1)	
Cognitive impairment		8 (5)				
Dizziness		12 (8)		13 (9)		
Dry mouth	1 (10)	18 (12)		6 (4)		
Dysgeusia		3 (2)				
Elevated blood pressure	1 (10)	11 (8)		13 (9)		
Fatigue			4 (2)		7 (5)	9 (4)
Gastrointestinal		28 (19)	144 (95)	52 (36)	94 (71)	184 (76)
Headache	1 (10)	12 (8)	3 (2)	17 (12)	5 (4)	7 (3)
Hypoglycemia					2 (1)	5 (2)
Hypokalemia		1 (1)				
Increased heart rate	3 (30)	9 (6)			2 (2)	2 (1)
Injection site reaction					4 (3)	1 (0.41)
Insomnia/Sleep disorder	1 (10)	18 (12)		12 (8)		
Metabolic acidosis	1					
Other neuropsychiatric symptoms	1 (10)		1 (1)	26 (18)	1 (1)	3 (1)
Palpitations	2 (20)	12 (8)				
Paresthesia		11 (7)				
Renal impairment					2 (1)	
Seizure				1 (1)		
Suicidal ideation/Suicide attempts behavior		3 (2)		3 (2)	2 (2)	2 (1)
WMM discontinuation						
Yes, *n* (%)^a^	80 (73)	327 (67)	350 (80)	234 (75)	194 (56)	247 (43)
Discontinuation secondary to ADE, *n* (%)	13 (12)	102 (21)	115 (26)	89 (29)	51 (15)	81 (14)
Discontinuation secondary to inefficacy/Goal not achieved, *n* (%)	21 (19)	90 (18)	104 (24)	68 (22)	53 (15)	37 (6)
Lost to follow up, *n* (%)	18 (16)	47 (10)	35 (8)	19 (6)	21 (6)	14 (2)
Shortage/Backorder, *n* (%)^b^	N/A	N/A	N/A	N/A	N/A	59 (10)
Non‐adherent, *n* (%)	6 (5)	32 (7)	30 (7)	15 (5)	16 (5)	10 (2)
Reason not documented, *n* (%)	23 (21)	39 (8)	41 (9)	25 (8)	15 (4)	9 (2)
Other, *n* (%)	18 (16)	69 (14)	70 (16)	51 (16)	69 (20)	34 (6)
No, *n* (%)	23 (21)	133 (27)	84 (19)	65 (21)	124 (36)	301 (53)
Yes, but re‐initiated, *n* (%)	4 (3)	25 (5)	5 (1)	11 (4	23 (6)	12 (2)
Not documented/Missing, *n* (%)	3 (3)	5 (1)	2 (∼0%)	1 (∼0%)	6 (2)	12 (2)
Duration of therapy, *n* (%)						
< 3 months	30 (28)	141 (29)	199 (46)	131 (42)	70 (20)	70 (12)
3 to < 6 months	31 (28)	85 (17)	90 (21)	58 (19)	58 (17)	67 (12)
6 to < 12 months	29 (27)	137 (28)	84 (19)	71 (23)	95 (27)	253 (44)
≥ 12 months	19 (17)	127 (26)	64 (15)	50 (16)	1124 (36)	179 (31)
Median number of weeks (IQR)	21 (12–38)	27 (12–50)	16 (7–36)	16 (6–36)	36 (16–60)	42 (24–54)
Adherence^c^						
Adherent, *n* (%)	57 (52)	307 (63)	193 (44)	159 (51)	226 (65)	434 (76)
Non‐adherent, *n* (%)	20 (18)	64 (13)	113 (25)	56 (18)	60 (17)	46 (8)
Not documented/missing, *n* (%)	33 (30)	119 (24)	135 (31)	96 (31)	61 (18)	92 (16)

*Note*: *N* = 2271 the unit of analysis for this table represents medication exposures since patients may have received one or more WMM during the MUE timeframe.

^a^ Multiple reasons may have been selected for WMM discontinuation.

^b^ Only evaluated for semaglutide.

^c^ Adherence was determined through documented provider assessment in progress notes. If no comments were found, the reviewers calculated a Medication Possession Ratio (MPR). An MPR of 80% or greater was determined to be adherent, while an MPR below 80% would indicate non‐adherence.

Considering the high proportion of patients with psychiatric comorbidities in the MUE cohort, subgroup analyses were conducted to evaluate the occurrence of certain mental health ADEs in patients with and without predisposing past medical histories. Specifically, we attempted to evaluate psychotic symptoms ADE in patients with and without a past medical history of schizophrenia spectrum and other unspecified psychotic disorders, as well as suicidal ideation/suicide attempt (SI/SA) while receiving WMM in patients with and without a history of SI/SA. However, due to the low number of events recorded, we were unable to derive meaningful findings.

Thirty‐six VAMCs participating in the MUE responded to the post‐MUE survey on local practices (Table 6). A common obstacle encountered was the semaglutide shortage during the MUE timeframe. The most common shortage management strategy was not permitting new‐starts (n = 20 sites) as per national VHA guidance. Fifty‐three percent of sites reported using off‐label semaglutide (OZEMPIC®) for weight loss alone. Sites responded that noticeable patient barriers to successful weight management included misunderstanding of the importance of continued lifestyle intervention (n = 15 sites), expectation of medication efficacy exceeding actual weight loss attained (n = 15 sites), and weight loss plateau (n = 13 sites). Among prescribing barriers, drug shortages (n = 20 sites), inadequate participation (n = 18 sites) or documentation (n = 11 sites) of CLI as required by CFU, patient preference (n = 11 sites), provider preference (n = 10 sites), and denied prior authorization (n = 8 sites) were identified.

**TABLE 6:** WMM MUE facility survey^a^.

Response from 36 sites on local practice measures	*N* (%)
Used national criteria for use of WMMs only (no additional local restrictions or criteria)	25 (69)
Observed provider off‐label use of semaglutide (OZEMPIC®)	19 (53)
Most common management strategies for semaglutide drug shortage	
No new starts	20 (56)
Transition to different GLP‐1 agonist	12 (33)
Transition to non GLP‐1 agonist	11 (31)
Hold drug until shortage resolved	11 (31)
Observed patient‐specific barriers to successful weight management	
Importance of continued participation in CLI not understood	15 (42)
Patient's expectation of medication efficacy greater than actual efficacy	15 (42)
Patient did not understand that weight loss would eventually plateau (*n* = 13)	13 (36)
Patient did not realize need to take WMM on chronic basis to maintain weight loss	5 (14)
Observed prescribing process barriers to successful weight management	
Drug shortages	20 (56)
Inadequate participation in CLI to meet criteria for use	18 (50)
Inadequate documentation of CLI to meet criteria for use	11 (31)
Patient preference not aligned with criteria for use	11 (31)
Provider preference not aligned with criteria for use	10 (28)
Prior authorization request denied	8 (22)
Planned educational efforts to optimize WMM prescribing based on MUE	
Patient education	5 (14)
Provider education	11 (31)
Develop academic detailing efforts/materials for weight management	11 (31)

^a^ Sites may select multiple responses for each question.

## 4 DISCUSSION

Limited data are available regarding the applicability of tightly controlled clinical trials to the Veteran patient population.^3^, ^4^, ^5^ Our evaluation of the six WMMs provides additional information on treatment selection, patient response, adherence and ADEs in a representative population of Veterans receiving care across 37 VA medical centers.

Weight reduction consistent with the hierarchy of benefit based on published literature was observed with each of the evaluated WMMs. However, the extent of weight reduction, or percent of the population with clinically meaningful weight reduction of at least 5%, did not reflect those reported in clinical trials, whereas the discontinuation rate due to an ADE was higher in this MUE cohort (Table 7).^13^, ^20^

**TABLE 7:** Comparison of observed results and published literature.

Chronic weight management medication	≥5% weight loss		Discontinuation due to an adverse event	
	Clinical trial data	MUE results (95% CI)	Clinical trial data	MUE results (95% CI)
Naltrexone/bupropion	55%^16^	28% (22.45%, 35.03%)	12%^16^	29% (23.66%, 33.99%)
Orlistat	45%^16^	17% (12.23%, 21.92%)	10%^16^	26% (22.04%, 30.44%)
Phentermine/topiramate	74%^16^	43% (37.26%, 48.69%)	10%^16^	21% (17.30%, 24.68%)
Liraglutide (SAXENDA®)	63%^16^	42% (34.93%, 48.80%)	13%^16^	15% (11.14%, 18.87%)
Semaglutide (WEGOVY®)	86%^8^	62% (56.31%, 66.62%)	7%^8^	14% (11.41%, 17.29%)

In general, reported ADEs were similar to those in published information. Limited data exists with the use of WMMs in patients with a history of mental illness including major depressive disorder, other serious psychiatric disorders, or history of suicidal attempt or recent suicidal behavior or ideation, as these conditions were excluded in clinical trials. It is therefore important to note the high percentage of patients in the MUE cohort with psychiatric comorbidity at baseline (61%), indicative of the high prevalence of mental illness among Veterans. The MUE specifically evaluated ADEs of psychotic symptoms reported during use of the WMM in a cohort of patients with and without a past medical history of schizophrenia spectrum and other unspecified psychotic disorders, as well as reports of suicidal attempts or ideation while receiving the WMM in a cohort of patients with or without a history of suicidal attempt or ideation. No safety signals surfaced from this evaluation. This may be due to the small number in each subgroup as well as prescriber avoidance of higher‐risk medications in patients with psychiatric conditions.

At the time of the MUE, naltrexone/bupropion, orlistat, and phentermine/topiramate were on the VA National Formulary (VANF) with prior authorization based on national CFU. VANF is a dynamic list of all medications available throughout the entire VA system. While items listed on the VANF are covered by VA pharmacy benefits, products with a prior authorization require additional review by an adjudication body. The national CFU guidance assists practitioners in clinical decision‐making to standardize and improve the quality of patient care. Liraglutide (SAXENDA®) and semaglutide (WEGOVY®) were available on a non‐formulary basis in accordance with the national CFU. Phentermine was available as a non‐formulary agent without national CFU. In general, the VANF medications are to be considered prior to non‐formulary agents. This was reflected in the high percentage of first line use of naltrexone/bupropion (86%), orlistat (88%), and phentermine/topiramate (90%). The relatively high use of first‐line therapy with the non‐formulary agents liraglutide (SAXENDA®) (74%) and semaglutide (WEGOVY®; and off‐label use of VANF Ozempic®) (70%) is likely due to satisfying criteria for first‐line therapy based on BMI or other comorbidities. These prescribing patterns were used to inform the discussion of the VA National Formulary Committee on projected utilization and formulary management.

Within VA, participation in CLI is a criterion for the use of the WMMs and is primarily achieved through the MOVE! program. According to the MUE results, 88% of patients were documented as having attended MOVE! or another approved CLI. These patients had a greater proportion who achieved a ≥5% weight reduction when compared to those who did not (49% vs. 37%).

In this MUE, 79% of patients treated with WMMs were within Class II (severe) or III (more severe) obesity at baseline. This may reflect providers prioritizing medication management in these higher classes of obesity. Additionally, patients with elevated BMIs were more likely candidates for first‐line therapy with the in‐demand GLP‐1 agonist medication.

We observed a large proportion of patients who discontinued therapy prior to 3 months. A plausible explanation may be attributed to the Veteran's weakened adherence from a perceived lack of efficacy during the early months. This was observed in a 2024 cohort study, which found a 6% increase in odds of remaining adherent if there was a 1% weight loss improvement at 6 months.^23^

The post‐MUE site survey provided insight for guiding future VA academic detailing efforts. VA Academic Detailing is an internal program that supports VA health care professionals through education on current, evidence‐based treatments and resources to ensure consistent, high‐quality care across all sites.^24^ The survey underscored the importance of patient communication regarding realistic expectations for weight loss and necessary long‐term therapy commitments as well as the importance of CLI. The prescribing barriers were shared with the VA National Formulary Committee, MOVE!, and VA Academic Detailing leadership. This resulted in several of these barriers being addressed in the Weight Management Academic Detailing materials. Moreover, a frequently asked questions (FAQ) document was developed in collaboration with MOVE! to address some of the perceived barriers to prescribing WMMs related to the criteria for patient participation in a CLI.

Results of the MUE have directed discussions of projected utilization and formulary management as well as areas of focus for continued safety monitoring, and the importance of addressing barriers through patient and provider education. Initiatives for integrated weight management teams, incorporating CLI, appropriate use and follow‐up of WMMs, and patient and provider education are encouraged at the local level. In addition to evaluating newly approved WMMs, future MUE projects will focus on a large database evaluation of suicidal ideation and/or attempts, and other severe or acute adverse events in the larger WMM prescribed population. This will provide additional safety insight for Veteran patients in light of a recent retrospective study showing a lower risk of incident and recurrent suicidal ideation in patients receiving semaglutide compared with other non GLP‐1 agonist medications.^25^

Inherent limitations exist with retrospective chart reviews. Data used for evaluating safety and effectiveness are dependent on the completeness of provider documentation in the electronic health record (EHR). Incomplete or missing documentation may not necessarily equate to a true absence of observation. Conversely, affirmative responses from chart review may underestimate an observation not documented in the EHR. One area of missing data was documented weights. Due to the increase in telehealth visits that resulted from the SARS‐CoV‐2 pandemic, both self‐reported and clinic‐measured weights were collected. However, weight changes were calculated only for paired methods (i.e., both self‐reported or both clinic‐measured). Furthermore, patients were excluded due to absence of a documented baseline weight. These exclusions may have introduced bias by excluding sporadic users of the VA system. However, since patients often had more than one reason for exclusion, only 151 more patients would have qualified if the criterion for baseline weight was omitted. Inherent to MUE operations activity and unlike research studies, unmeasured biases are likely built into clinical practice, such as providers avoiding certain higher‐risk medications in patients with mental health history or prior social habits. This could result in fewer events of interest with the higher‐risk medications. Another source of potential bias is the VA GLP‐1 agonist CFU requiring intolerance or inadequate response to two prior formulary agents. Providers may have a lower threshold for attributing adverse events to formulary agents to qualify a patient more expediently for a GLP‐1 agonist, thus resulting in a higher rate of reported ADEs for formulary agents.

The findings of this evaluation may have also been impacted by challenges related to the SARS‐CoV‐2 pandemic. The shelter‐in‐place orders may have led to decreased physical activity and excess weight gain.^26^, ^27^ The pandemic also affected healthcare utilization patterns with patients delaying or avoiding non‐urgent clinic visits.^28^ These changes in lifestyle and routine healthcare visits may have affected the documentation of BMIs and reduced the number of eligible patients.

Caution should be exercised in making comparisons between WMMs as the findings reflect how these WMMs performed as used within the VA system. Existing biases as well as lack of randomization and controls prevent a direct comparison of effectiveness and safety outcomes between WMMs. The prescribing patterns and observed trends in safety and effectiveness, however, can be used as data to support planning for future use of each WMM.

## 5 CONCLUSION

This MUE presents practical insight regarding the safety and weight loss potential of WMMs among the older and predominantly male Veteran population. We found a lower percentage of weight loss and a higher discontinuation rate due to ADEs compared with those reported in clinical trials. However, Veterans experienced beneficial weight loss, particularly with semaglutide, phentermine/topiramate, and liraglutide. Therefore, the MUE data allow for better assessment of benefits and risks for Veterans prescribed WMMs and underscore the need for improved patient and provider education regarding therapy expectations and the importance of CLI. Further plans will include safety monitoring with a focus on psychiatric and other ADEs, improvements in processes such as appropriate and equitable prescribing of first line GLP‐1 agonists, and evaluation of new WMMs.

## AUTHOR CONTRIBUTIONS

**Samantha Walczuk** ‐ Conducted the project, educated chart reviewers, interpreted data, drafted manuscript. **Muriel Burk** ‐ Managed the project, developed protocol, designed data abstraction tool, coordinated efforts with participant sites, interpreted data, and drafted manuscript. **Elaine Furmaga** ‐ Served as a clinical consultant and subject matter expert, developed the MUE protocol, interpreted data, and contributed to manuscript write‐up. **Samaneh Ghassemi** ‐ Prepared manuscript for submission, interpreted data, served as liaison for pharmacovigilance. **Madeline McCarren** ‐ Served as a biostatistician, conducted data analysis, interpreted data, and reviewed the manuscript. **Kenneth Bukowski** ‐ Data programmer, technical support. **Peter Glassman** ‐ Project inception, design of MUE, clinical consultant, manuscript review. **Fran Cunningham** ‐ Project oversight and advisor, design of MUE, manuscript review, served as consultant for pharmacovigilance.

## CONFLICT OF INTEREST STATEMENT

The authors declare no conflicts of interest.
